# Health-related quality of life of Adolescent and Young Adult Cancer Survivors before and during the COVID-19 pandemic: longitudinal improvements on social functioning and fatigue

**DOI:** 10.1186/s41687-023-00629-0

**Published:** 2023-09-13

**Authors:** Carla Vlooswijk, Silvie H. M. Janssen, Lonneke V. van de Poll-Franse, Rhodé Bijlsma, Suzanne E. J. Kaal, J. Martijn Kerst, Jacqueline M. Tromp, Monique E. M. M. Bos, Roy I. Lalisang, Janine Nuver, Mathilde C. M. Kouwenhoven, Winette T. A. van der Graaf, Olga Husson

**Affiliations:** 1 Research and Development, Netherlands Comprehensive Cancer Organisation, 3511 DT Utrecht, The Netherlands; 2 Department of Psychosocial Research and Epidemiology, Netherlands Cancer Institute, 1066 CX Amsterdam, The Netherlands; 3 Department of Medical Oncology, Netherlands Cancer Institute-Antoni Van Leeuwenhoek, 1066 CX Amsterdam, The Netherlands; 4 Department of Medical and Clinical Psychology, Tilburg University, 5037 AB Tilburg, The Netherlands; 5 Department of Medical Oncology, University Medical Center Utrecht, 3584 CX Utrecht, The Netherlands; 6 Department of Medical Oncology, Radboud University Medical Center, 6525 GA Nijmegen, The Netherlands; 7 Department of Medical Oncology, Amsterdam University Medical Centers, 1105 AZ Amsterdam, The Netherlands; 8 Department of Medical Oncology, Erasmus MC Cancer Institute, Erasmus University Medical Center, 3015 GD Rotterdam, The Netherlands; 9 Department of Internal Medicine, Division of Medical Oncology, GROW-School of Oncology and Reproduction, Maastricht UMC+ Comprehensive Cancer Center, 6229 HX Maastricht, The Netherlands; 10 Department of Medical Oncology, University Medical Center Groningen, 9713 GZ Groningen, The Netherlands; 11 Department of Neurology, Amsterdam UMC, Amsterdam University Medical Centers, Location VUmc, 1081 HV Amsterdam, The Netherlands; 12 Department of Surgical Oncology, Erasmus MC Cancer Institute, Erasmus University Medical Center, 3015 GD Rotterdam, The Netherlands

**Keywords:** Adolescents and young adults, COVID-19, Health-related quality of life, Social functioning, Survivorship, Cancer

## Abstract

The health-related quality of life (HRQoL) among long-term Adolescent and Young Adult Cancer Survivors (AYACS) and an age- and sex-matched normative population was examined. Although the HRQoL of AYACS was worse compared to the normative population before and during the COVID-19 pandemic, the scores of AYACS improved over time in contrast to the normative population. Presumably, AYACS are used to adjusting their lives to stressful life events. Furthermore, the lockdown may have been beneficial for AYACS who face difficulties fully participating in society due to the impact of cancer. AYACS who encounter HRQoL issues could benefit from support interventions to empower them and build resilience.

**Supplementary Information:**

The online version contains supplementary material available at 10.1186/s41687-023-00629-0.

## Introduction

The coronavirus disease 2019 (COVID-19) pandemic has had a great impact on the capacity of patients and healthcare systems. At the peaks of the pandemic, many appointments and treatments were cancelled or postponed. There is emerging research on the psychological impact of COVID-19 on the general population, but especially on vulnerable populations such as cancer patients [1–7].

Adolescent and Young Adult cancer survivors (AYACS), in this manuscript defined as adolescents and young adults who had been diagnosed with primary cancer between ages 18–39 years, might be particularly vulnerable to the effects of the pandemic, because of their unique developmental, educational, social and emotional needs [8]. Studies conducted before the start of the COVID-19 pandemic show that AYACS experience a lower health-related quality of life (HRQoL) compared to the general population [9–11]. AYACS already face significant challenges and psychological distress due to a cancer diagnosis, which are possibly exacerbated by COVID-19.

Cancer patients in general have a higher risk of developing complications from COVID-19, which can be partly ascribed to factors such as older age, higher smoking rates, comorbidities, frequent healthcare exposures and the effects of cancer therapies [4]. During the COVID-19 pandemic, cancer patients also experienced more mental health concerns as well as an enduring sense of fear and worry about their potential health risks and changes in their follow-up care [5]. Furthermore, quarantine and social isolation contribute to feelings of isolation and loneliness among cancer patients which further compromised their mental health [2, 6, 7]. Studies among AYACS investigating the impact of the COVID-19 pandemic show high levels of insomnia, loneliness and psychological distress, with feelings of anxiety reported more prominently than depressive symptoms [12–14].

Previously conducted studies on the impact of COVID-19 on AYACS focus on adolescents and young adults who had active treatment or were within 5 years after diagnosis. To the best of our knowledge, there are no existing studies that investigate the impact of the COVID-19 pandemic on the HRQoL among long-term (5 or more years) AYACS. Moreover, other studies do not have a longitudinal research design and do not include data of an age- and sex-matched normative population. Insight into the impact of the COVID-19 pandemic on HRQoL of long-term AYACS is warranted. Understanding the unique challenges that this population may experience currently as a result of the COVID-19 pandemic is necessary in order to support and treat AYACS optimally. Therefore, within a subsample of the SURVAYA study [15], we investigate the differences in HRQoL between AYACS and an age- and sex-matched normative population (1) before-, (2) during the COVID-19 pandemic and (3) over time.

## Methods

### Setting and population

The cross-sectional population-based SURVAYA study was conducted among long-term survivors (5–20 years) of cancer on AYA-age (18–39 years) [15]. They received primary cancer treatment between 1999 and 2015 and were treated at the Netherlands Cancer Institute/Antoni van Leeuwenhoek Hospital or at an academic hospital in the Netherlands.. The SURVAYA study was approved by the Institutional Review Board of the Netherlands Cancer Institute (IRBd20-115) and registered within clinical trial registration (NCT05379387).

### Data collection

Data was collected between May 2019 and June 2021 and the data collection process is described in more detail elsewhere [15]. Patients who participated in the SURVAYA study before the start of the first COVID-19 lockdown (March 23, 2020), and who gave consent to be invited for additional questionnaire studies (n = 1089), were invited to complete a COVID-19 specific questionnaire between 16 April and 14 May 2020 (Additional file 1: Fig. S1).

### Normative population

The normative population was derived from a reference cohort of the general Dutch population (CentER panel), which is representative for the Dutch-speaking population in the Netherlands [16]. Members of this panel received online questionnaires in November 2017 (wave 1) and May 2020 (wave 2) (n = 907). This normative population was matched based on the age and sex distribution of the SURVAYA-COVID-19 study. A frequency matching method was used with age strata (18–30 years, 30–40 years and 40–60 years) and sex strata (females and males) to maximize the number of control participants matched to AYACS. This resulted in 108 matched cancer-free individuals for the 407 AYACS.

### Study measures

Self-reported sociodemographic data included sex, marital status (with or without a partner) and educational level (no education or primary education, secondary (vocational) education and higher (vocational and University education). Linkage with the Netherlands Cancer Registry (NCR) provided information on age at questionnaire completion, time since diagnosis, type of cancer, tumor stage and primary treatment modality (surgery, chemotherapy, radiotherapy, hormone therapy, targeted therapy and stem cell therapy). HRQoL was assessed with the European Organisation for Research and Treatment of Cancer Quality of Life Questionnaire (EORTC QLQ-C30) [17]. Linear transformation was used to obtain scores ranging from 0 to 100, where a higher score represents a higher (“better”) level of functioning (physical functioning, role functioning, emotional functioning, cognitive functioning, social functioning and global quality of life) or higher (“worse”) level of symptoms (fatigue, pain, dyspnea and insomnia). Clinically relevant differences were determined using the evidence-based guidelines for interpretation of the EORTC QLQ-C30. These guidelines define a minimum number of points that are required to detect a clinically relevant difference [18, 19]. Thresholds of clinical importance were used for social functioning (58), cognitive functioning (75), fatigue (39) and pain (25) to identify patients with impaired/within normal functioning before and during the COVID-19 pandemic [20].

### Statistical analyses

All statistical analyses were performed using SAS (version 9.3 for Windows; SAS institute Inc., Cary, NC). Differences between socio-demographic and clinical characteristics between AYACS and the normative population were assessed with a chi-square test (categorial variables) or independent sample t-test (continue variables). The HRQoL mean changes of AYACS and normative population over time were assessed with paired sample t-tests. Analyses of covariance (ANCOVA) were carried out to compare HRQoL between AYACS and the normative population, adjusted for educational level (high-medium/low) and partner status (yes/no). Due to the multiple statistical tests conducted in this study and to avoid type 1 errors, all differences with a P-value < 0.01 were indicated as statistically significant. A graphical representation of the intraindividual longitudinal changes over time is displayed for HRQoL scales that showed medium and small clinically relevant differences.

## Results

### Sample characteristics

In the SURVAYA study, 4010 AYACS participated, of whom 1089 participated before the COVID-19 pandemic and were eligible to participate in the additional COVID-19 questionnaire study (Additional file 1: Fig. S1). In the additional COVID-19 questionnaire study, 407 AYACS participated (37%). The demographic and clinical characteristics of the included AYACS (n = 407) and age- and sex-matched normative population (n = 108) are summarized in Table 1. AYACS were significantly more likely to be married/partnered (p < 0.0001) and were higher educated (p < 0.0001) than the matched normative population.

**Table 1:** Socio-demographic and clinical characteristics of AYA cancer survivors and matched normative population

		AYA cancer survivors (N = 407)	Matched normative Population (N = 108)	P value
		n (%)	n (%)	
Gender	Male	129 (31.7%)	34 (31.5%)	0.9
	Female	278 (68.3%)	74 (68.5%)	
Age (at time of questionnaire), (mean (SD) in years)		45.5 (6.9)	44.4 (10.3)	0.2
	23–40 years	78 (19.2%)	21 (19.4%)	
	40–50 years	220 (54.1%)	58 (53.7%)	
	50–60 years	109 (26.8%)	29 (26.9%)	
Married/partnered	Partner	334 (82.1%)	59 (54.6%)	< .00011
Education level	No education or primary education	2 (0.5%)	4 (3.7%)	< .00011
	Secondary (vocational) education	117 (28.7%)	71 (66.36%)	
	Higher (vocational) and University education	288 (70.8%)	32 (29.9%)	
Treatment/follow up status	Currently being treated or have to start treatment	16 (3.9%)	NA	
	Completed treatment, now in follow-up	194 (47.7%)		
	Completed treatment, not in follow-up	197 (48.4%)		
Time since diagnosis, (mean (SD) in years)		12.4 (4.3)	NA	
	5–10 years	131 (32.2%)		
	≥ 11–15 years	151 (37.1%)		
	≥ 16–20 years	125 (30.7%)		
Type of cancer	Bone and soft tissue sarcoma	19 (4.7%)	NA	
	Melanoma	24 (5.9%)		
	Colon and rectum	11 (2.7%)		
	Breast	153 (37.6%)		
	Female genitalia	29 (7.1%)		
	Germ cell tumors	55 (13.5%)		
	Lymphoid haematological malignancies	44 (10.8%)		
	Myeloid hematological malignancies	13 (3.2%)		
	Thyroid gland	15 (3.7%)		
	Central nervous system	22 (5.4%)		
	Other^3^	22 (5.3%)		
Primary treatment modality	Surgery	338 (83.0%)	NA	
	Chemotherapy	237 (58.2%)		
	Radiotherapy	231 (56.8%)		
	Hormone therapy	84 (20.6%)		
	Targeted therapy	40 (9.8%)		
	Stem cell therapy	14 (3.4%)		

^1^Chi-square p-value, ^2^Independent sample t-test, ^3^Head and neck: 5 (1.2%); Digestive tract, other: 4 (1.0%); Respiratory tract: 6 (1.5%); Male genitalia: 1 (0.2%); Urinary tract: 5 (1.2%); Eye: 1 (0.2%)

### HRQoL before and during COVID-19 pandemic

**Fig. 1:**
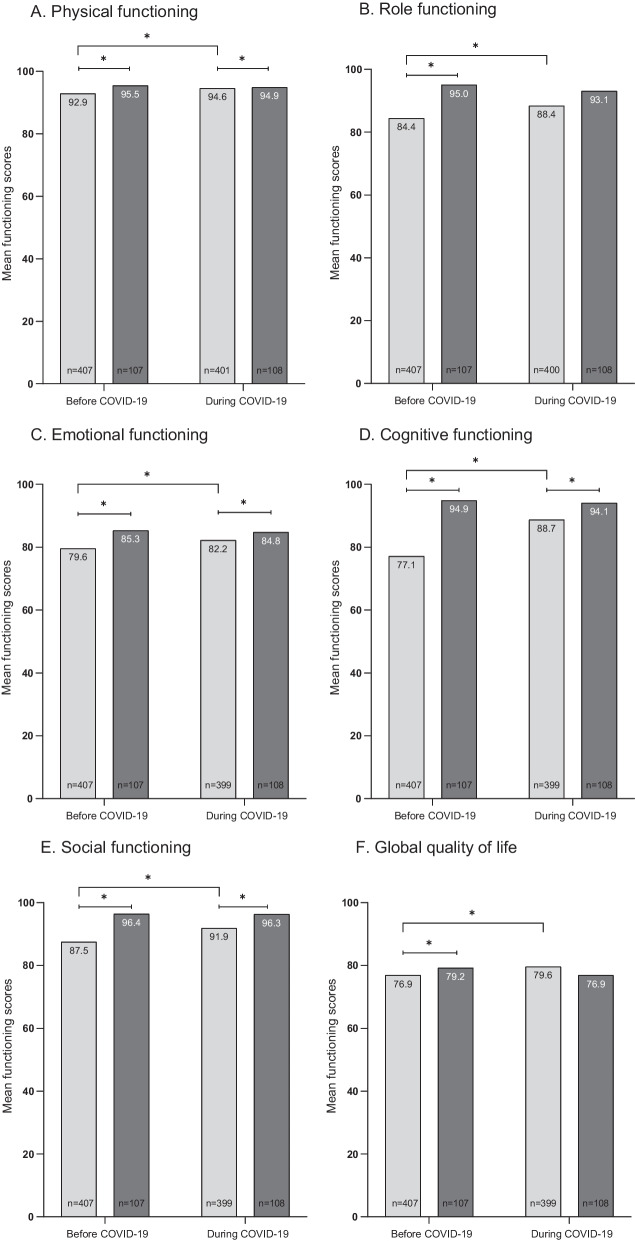

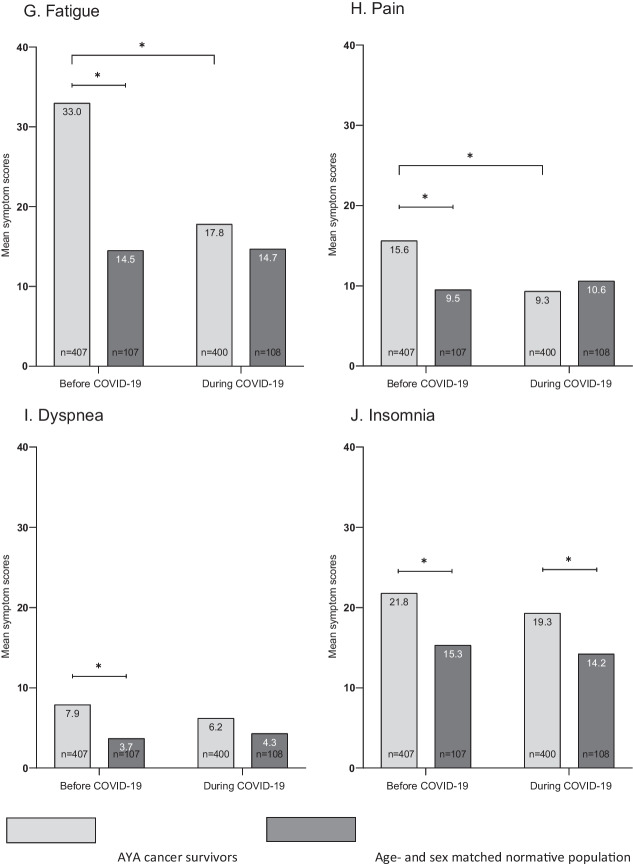
**A**–**J** HRQoL scores of AYACS and age- and sex-matched normative population before and during the COVID-19 pandemic. *Note*. A higher score on the functioning scales refers to a better HRQoL (physical functioning, role functioning, emotional functioning, cognitive functioning, social functioning and global quality of life). A higher score on symptoms scales refers to more symptoms (fatigue, pain, dyspnea and insomnia). *Significant difference (p < 0.001)

Before COVID-19, compared to an age- and sex-matched normative population, AYACS reported on average, worse scores on the EORTC QLQ-C30 functioning and symptoms scales (Fig. 1A–J). These differences between AYACS and the normative population were considered of medium clinical relevance for fatigue (− 18.5 score points, p-value: < 0.0001) and small for role-functioning (+ 10.6 score points, p-value: < 0.0001), social-functioning (+ 8.9 score points, p-value: < 0.0001), pain (− 6.1 score points, p-value: < 0.0001), dyspnea (− 4.2 score points, p-value: 0.0079) and insomnia (− 6.5 score points, p-value: 0.00554).

During the COVID-19 pandemic, physical-functioning, emotional-functioning, cognitive-functioning, social-functioning, and insomnia scores of AYACS were significantly worse compared to the normative population. However, these differences were smaller than before the start of the COVID-19 pandemic. During the COVID-19 pandemic, only a small clinically relevant difference was found for cognitive functioning (+ 5.4 score points, p-value: 0.0004) and insomnia (− 5.1 score points, p-value: 0.0098) between AYACS and the normative population.

**Fig. 2:**
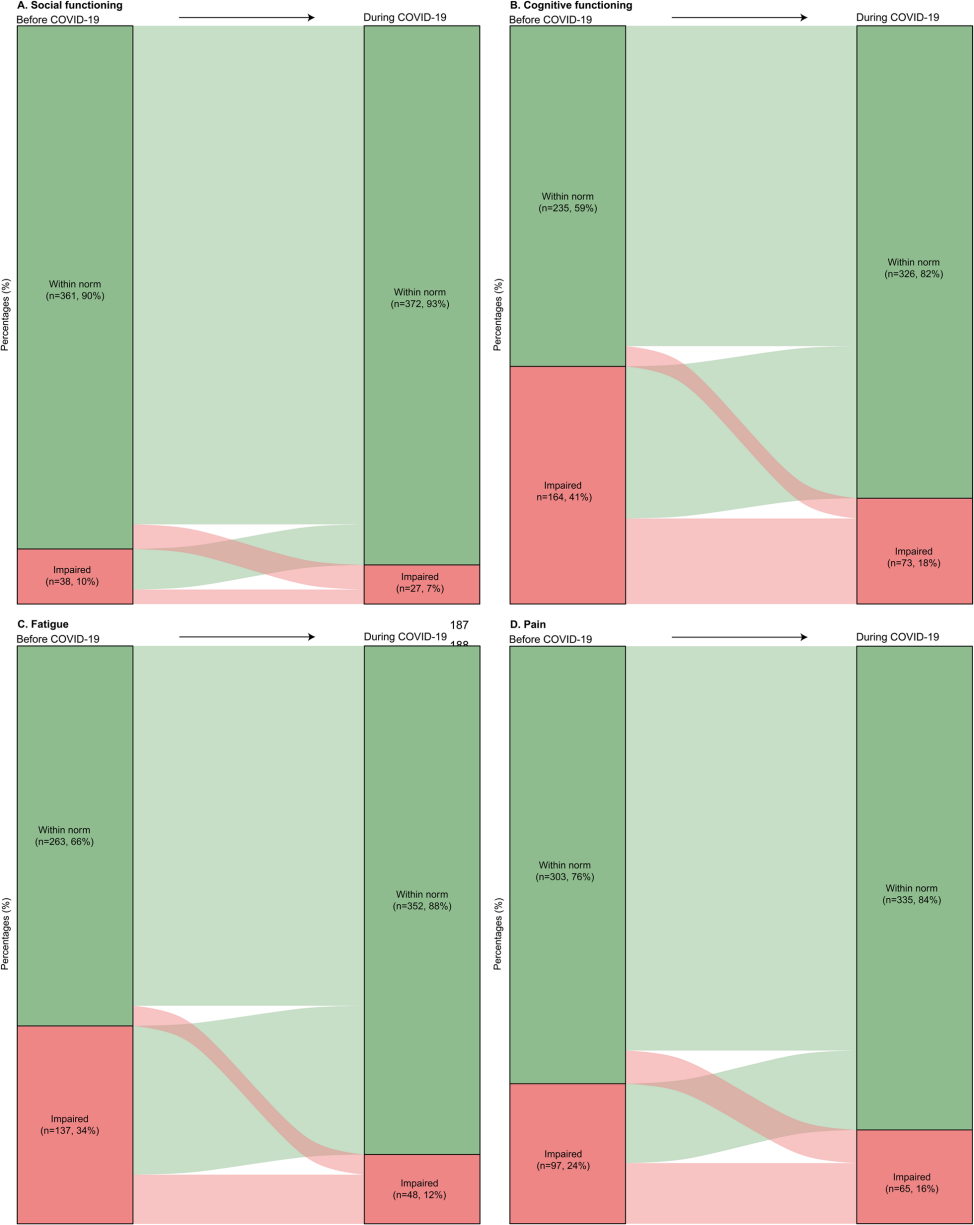
**A**–**D** Graphical representation of intraindividual longitudinal changes in **A** social functioning, **B** cognitive functioning, **C** fatigue and **D** pain among AYACS. *Note*. **A** Within norm (before COVID-19) to within norm (during COVID-19): 344 AYACS (86%); Within norm (before COVID-19) to impaired (during COVID-19): 17 AYACS (4%); Impaired (before COVID-19) to within norm (during COVID-19): 28 AYACS (7%); Impaired (before COVID-19) to impaired (during COVID-19): 10 (3%). **B** Within norm (before COVID-19) to within norm (during COVID-19): 221 AYACS (55%); Within norm (before COVID-19) to impaired (during COVID-19): 14 AYACS (4%); Impaired (before COVID-19) to within norm (during COVID-19): 105 AYACS (26%); Impaired (before COVID-19) to impaired (during COVID-19): 59 (15%). **C** Within norm (before COVID-19) to within norm (during COVID-19): 249 AYACS (62%); Within norm (before COVID-19) to impaired (during COVID-19): 14 AYACS (4%); Impaired (before COVID-19) to within norm (during COVID-19): 103 AYACS (26%); Impaired (before COVID-19) to impaired (during COVID-19): 34 (9%). **D** Within norm (before COVID-19) to within norm (during COVID-19): 280 AYACS (70%); Within norm (before COVID-19) to impaired (during COVID-19): 23 AYACS (6%); Impaired (before COVID-19) to within norm (during COVID-19): 55 AYACS (14%); Impaired (before COVID-19) to impaired (during COVID-19): 42 (11%)

AYACS reported better HRQoL scores during the COVID-19 pandemic than before its start. The improvements in cognitive functioning (+ 11.6 score points, p-value: < 0.0001) and fatigue (− 15.2 score points, p-value: < 0.0001) were of medium clinical relevance. Differences in social functioning (+ 4.4 score points, p-value: < 0.0001) and pain (− 6.3 score points, p-value: < 0.0001) were considered of small clinical relevance. The HRQoL scores of the normative population remained stable over time. Figure 2A–D shows the intraindividual longitudinal changes in social-functioning, cognitive-functioning, fatigue and pain among AYACS.

## Discussion

This study aimed to investigate the longitudinal impact of the COVID-19 pandemic on the HRQoL of long-term AYACS and to compare their HRQoL with the general population before and during the pandemic. AYACS experienced worse HRQoL scores, before and during the COVID-19 pandemic compared to the normative population. However, in contrast to the normative population, the HRQoL of AYACS improved during the pandemic with the largest differences in social functioning, cognitive functioning, fatigue and pain.

Our results showed that long-term AYACS experienced better HRQoL scores during the pandemic than before it. To the best of our knowledge, no studies exist regarding the impact of COVID-19 on HRQoL in long-term AYACS. There are several studies with a cross-sectional study design among adolescents and young adults in active treatment or shortly after diagnosis that investigate the impact of the COVID-19 pandemic on psychological well-being. High levels of psychological distress during the COVID-19 pandemic were reported [12, 14, 21]. Specifically, the cross-sectional study of Košir et al. found that 30% of the adolescents and young adults experienced more psychological distress and 60% reported more anxious feelings during the COVID-19 pandemic than previously [14]. Moreover, adolescents and young adults who were undergoing treatment or were within 6 months of treatment completion reported higher levels of psychological distress on average. This was supported by a study where the lowest elevated psychological distress was found in those off treatment, for whom healthcare was also the least affected by the pandemic [14]. Adolescents and young adults who are undergoing treatment likely experience more distress than long-term AYACS due to uncertainty about beginning or continuing treatment under the COVID-19 restrictions during treatment in the first five years after cancer diagnosis.

In the long-term, AYACS reported worse HRQoL outcomes in all HRQoL domains overall compared to the general population. This is in line with results from a review by Quinn et al. and other recent international studies [9, 10, 22, 23]. However, our study found no adverse impact on HRQoL attributed to COVID-19 specifically, even though cancer survivors are more at risk of developing serious illnesses from the disease. Two possible explanations are that the ability of AYACS to deal with abnormal life-situations is obviously already high and better than their peers. A study by Jacobsen et al. found that high levels of resilience acted as protective factor for psychological distress in AYACS with cancer during the COVID-19 pandemic [13]. Another explanation could be that some AYACS experienced more difficulties (e.g. stress because they were not able to work the required hours or manage all the work because of cognitive or physical problems) to participate fully in society before COVID-19. The societal changes that were implemented by governments to decrease the spread of highly infectious COVID-19, including travel restrictions and nationwide quarantine, resulted in a lockdown of society. This shut down could have been beneficial for those who struggled to fully participate in society just like their peers, such as those who experience the long-term physical and mental effects of cancer.

### Future implications

Considering that the HRQoL reported by long-term AYACS was still lower than that of the normative population, there are fruitful avenues for future research and interventions in AYA care are needed. Psychological and physical support is important for those at risk of poor HRQoL scores, even 5–20 years after diagnosis. To assess whether there are differences in HRQoL between subgroups of AYACS, additional analyses of HRQoL outcomes in a larger sample of AYACS would enable greater insight into the characteristics of those AYACs at greater risk of poor HRQoL outcomes. Analysing subgroups of AYACS stratified by age, education level, time since diagnosis and cancer type can inform health care services by informing the development and planning of interventions that take the needs of AYACS into consideration. Psychological and physical support interventions must address empowerment and building resilience to help AYACS live with the short- and long-term effects of cancer.

### Strengths and limitations

The interpretation of the findings of this study must consider some limitations. Among the SURVAYA respondents, participants were asked to complete the COVID-19 specific questionnaire study. Selection bias was reported within the SURVAYA study, since study participation was significantly lower among specific subgroups, such as males and survivors with a lower socio-economic status [15]. Non-response may have caused selection bias, limiting the generalizability of the results this study. Moreover, in this study, the AYACS study population obtained higher levels of education compared to the normative population and were more likely to be married/partnered. Previous research has shown that cancer survivors who obtained lower levels of education and/or cancer survivors who are unmarried or do not have a partner tend to have worse HRQoL outcomes and experience more distress [24, 25]. Therefore, this may have influenced our results and HRQoL scores of the AYACS might be overestimated in the cross-sectional comparisons between AYACS and normative population.

## Conclusion

Compared to an age- and sex matched normative population, AYACS experience worse HRQoL scores, both before and during the COVID-19 pandemic. However, the HRQoL of AYACS improved during the pandemic in contrast to the matched normative population. The lockdown of the society could have been beneficial for AYACS who experience short-and long-term effects of cancer which can hinder full participation in society just like their peers. Moreover, AYACS may be more adept at coping with abnormal life-threatening situations than the general population.

### Supplementary Information

**Additional file 1. Fig. S1**: Flowchart of the data collection process of the SURVAYA-COVID-19 study.

## Data Availability

The datasets generated and/or analysed during the current study are not publicly available due to privacy issues but are available from the corresponding author on reasonable request.

## Abbreviations

AYACS: Adolescent and Young Adult Cancer Survivors
COVID-19: Coronavirus Disease 2019
EORTC QLQ-C30: European Organisation for Research and Treatment of Cancer Quality of Life Questionnaire C30
HRQoL: Health-related quality of life
