# Supplementary material for: Health-related quality of life of Adolescent and Young Adult Cancer Survivors before and during the COVID-19 pandemic: longitudinal improvements on social functioning and fatigue
Source: J Patient Rep Outcomes. 2023 Sep 13;7:93. doi: 10.1186/s41687-023-00629-0 (PMC10499718; doi:10.1186/s41687-023-00629-0)
Supplement: Supplementary file 1 — Additional file 1. Fig. S1: Flowchart of the data collection process of the SURVAYA-COVID-19 study. [file 41687_2023_629_MOESM1_ESM.docx]

**Additional file 1; Figure S1.** Flowchart of the data collection process of the SURVAYA-COVID-19 study.

SURVAYA study population (n=4010)

**Participated in SURVAYA study during COVID-19**

(March 2020—June 2021) (n=2675)

**Participated in SURVAYA study before COVID-19**

(May 2019—February 2020) (n=1335)

Gave no consent for follow-up questionnaire (n=62)

Participated on paper (n=112)

Not responded yet or not finished at time of selection (n=69)

Deceased (n=3)

Wave 1: before COVID-19

**Eligible to participate in COVID-19 specific questionnaire study**

(June 2019—February 2020) (n=1089)

Wave 2: during COVID-19

Invited to additional COVID-19 related questionnaire

**Participated in COVID-19 specific questionnaire study**

( 16 April—14 May 142020) (n=407)
